# Psychoeducation and motivational interviewing to reduce relapses and increase patients’ involvement in antipsychotic treatment: interventional study

**DOI:** 10.1192/bjb.2020.28

**Published:** 2020-12

**Authors:** Gabriella Bröms, Lindah Cahling, Anders Berntsson, Lars Öhrmalm

**Affiliations:** 1 Unit of Clinical Epidemiology, Centre for Pharmacoepidemiology, Karolinska Institutet, Stockholm, Sweden; 2 PRIMA Child and Adult Psychiatry, Stockholm, Sweden; 3 Department of Medicine, Solna, Karolinska Institutet, Stockholm, Sweden

**Keywords:** Motivational interviewing, psychoeducation, schizophrenia, antipsychotics, adherence

## Abstract

**Aims and method:**

To assess whether the combination of motivational interviewing and psychoeducation affects relapse rate and stimulates involvement of people with psychosis in their treatment. We conducted an interventional study including patients with schizophrenia or schizoaffective disorder treated with oral antipsychotics, without previous experience of long-acting injectable antipsychotics (LAIs). They were randomised to either psychoeducation with motivational interviewing or a control group. Hospital admissions 18 months before and after the intervention, and switches to LAIs 18 months after the intervention, were recorded.

**Results:**

The two groups each comprised 101 participants. Fourteen from the intervention group and seven from the control group switched to LAIs. Five in the intervention group instigated the switch themselves, compared with zero controls (*P* = 0.06). Fourteen in the intervention group were readmitted to hospital during follow-up, compared with 23 in the control group (*P* = 0.14).

**Clinical implications:**

Psychoeducation with motivational interviewing may increase patients' involvement in their treatment and reduce the relapse frequency.

Adherence to pharmacological treatment by people with psychotic disorders is one of the major challenges in psychiatry, and its lack is an important predictor of relapse, even with small gaps in treatment, regardless of formulation.^1,2^ However, in observational studies long-acting injectable antipsychotics (LAIs) reduce the risk of discontinuation when compared with oral treatment.^3^ Patients are seldom encouraged to participate in the choice of antipsychotic medication and formulation,^4^ which is unfortunate, as it is known that their attitude to treatment becomes more positive with increased knowledge and experience of the treatment.^5^ Psychoeducation alone is not enough to improve adherence,^6^ but a combination of psychoeducation and motivational interviewing seems to be more promising.^7,8^

We studied switches from oral antipsychotics to LAIs as a proxy for patients' involvement and adherence and hospital admissions as a proxy for relapse rate.

## Method

We included 202 people with schizophrenia and schizoaffective disorder on oral antipsychotic treatment and no earlier experience of LAIs who were part of an earlier study conducted by our group.^9^ In the earlier study, apart from the individuals on oral treatment included in this study, we also included people currently on LAIs and questioned all participants on their perceptions and knowledge of antipsychotics in general, using semi-structured interviews.

The participants in the current study were block-randomised by diagnosis (schizophrenia/schizoaffective disorder), gender and age into two separate groups: either the intervention with psychoeducation and motivational interviewing group or a comparison group with no intervention. Participants in the intervention group were enrolled on giving written informed consent to participate in an intervention and to grant access to their own medical chart. If an individual refused to participate or was excluded for another reason, their matched comparator was excluded as well. The study was approved by the Regional Ethical Review Board in Stockholm (ref. 2015/47-31).

### Psychoeducation

As part of our earlier study, participants in the intervention group underwent a single semi-structured interview by a study nurse specialised in mental health (L.C.), in which perceptions and knowledge of antipsychotic formulations were examined.^9^ In summary, the earlier study revealed that participants lacked knowledge regarding differences in plasma concentration, side-effects and risk of readmission to hospital between LAIs and oral treatment. At the end of the interview, the study nurse provided psychoeducation, discussing and correcting misconceptions of the differences between oral and LAI antipsychotics. The risk associated with gaps in medication was also discussed.

### Motivational interviewing

The semi-structured interview, which included questions on beliefs and attitudes to treatment, was followed by an adapted form of motivational interviewing based on the original principles of the technique.^10^ L.C. had received training from a professional trainer on motivational interviewing in a 3-day course prior to the study. Participants were encouraged to discuss their personal ideas and ambivalences regarding their illness and treatment. Focus was on relapse-preventing factors, quality of life, exploring their own personal goals and treatment strategies, with no preference for either oral or LAI antipsychotics.

### Follow-up

Data on admissions were extracted from the participants' medical records during the 18-months before and after the intervention. Participants were not called for extra visits during the follow-up, and clinical events were noted as they appeared in the medical charts. Hospital admission was considered a proxy for relapse. We observed the frequency of participants in each group with a hospital admission before and after intervention respectively. During the follow-up period, we also measured the number of participants who switched from oral to LAI antipsychotics. We noted whether the switch had been instigated by the participant, by clear indication of this in the medical chart, as opposed to it being described as a decision made by the treating psychiatrist or not described at all. The former was considered a proxy for increased patient involvement.

### Statistical analysis

Anonymised data were analysed using Prism 5.03 for Windows. Sample comparisons were made using Fisher's exact test for categorical variables.

## Results

In total, there were 101 participants in the intervention group and an equal numberof comparators. Characteristics for both groups were proportionately matched regarding gender (46% females) and diagnosis (70% schizophrenia). The median age was 50 years for both groups, but the ranges differed slightly (21–84 years and 24–79 years for the intervention group and the comparator group respectively).

### Switching

**Fig. 1:**
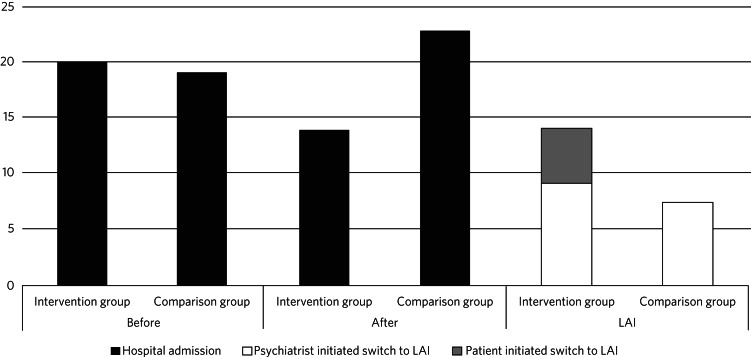
Hospital admissions before and after intervention and switches to a long-acting injectable antipsychotic (LAI).

After 18 months, 14 participants (14%) from the intervention group had switched to LAIs, compared with 7 (7%) in the comparison group (*P* = 0.17, Fig. 1). Five out of 101 participants (5%) in the intervention group suggested a switch themselves, compared with zero in the comparison group (*P* = 0.06). Regarding switching by decision of the psychiatrist, there was no significant difference between the groups (9 *v.* 7, *P* = 0.80).

### Hospital admission

In the intervention group, 20 participants (20%) were admitted to hospital at least once during the 18-month period before intervention, compared with 19 (19%) of the comparators (*P* = 1.0, Fig. 1). The numbers of participants admitted during the 18-month follow-up period after intervention were 14 (14%) and 23 (23%) respectively (*P* = 0.14).

## Discussion

In this interventional study, we found that a single session of psychoeducation and motivational interviewing in combination may promote patients' involvement in treatment choices of oral and LAI antipsychotics and reduce relapse frequency.

The higher number of participants switching to an LAI in the intervention group could reflect less resistance to changing their medication after psychoeducation and motivational interviewing. However, as at least five participants in the intervention group actively initiated the discussion of switching, the higher switching rate could also be explained by increased involvement in their treatment. This supports results from an earlier study by Barkhof et al, in which a targeted use of motivational interviewing seemed to improve medication adherence at least for certain groups of people with psychosis.^11^ Their adapted form of motivational interviewing had an active provision of psychoeducation, which makes it to some extent comparable to our interview, although their intervention included several sessions and included people with a recent relapse.

The number of participants admitted to hospital at least once in the 18-month period before intervention was comparable between groups, with only one more participant in the intervention group being admitted. In the follow-up period after intervention the corresponding analysis showed, however, nine fewer admissions in the intervention group. This could indicate an effect of psychoeducation and motivational interviewing on admission frequency, which in turn could be a result of increased adherence and involvement in antipsychotic treatment. This is in line with previous studies, which have reported positive effects of the combination of psychoeducation and motivational interviewing on adherence.^7,8,11^

Exploring patients' fears is a less frequently used technique, as reported in our earlier study, and participants in the same study lacked knowledge.^9^ Moreover, other research groups have reported reluctance among medical professionals to initiate the discussion of treatment.^4,12^ The possible positive effect of psychoeducation and motivational interviewing may encourage a more exploring approach in treatment discussion with the patient.

### Limitations

Our study has several limitations. Individuals with cognitive disorders and acute relapse at the time of inclusion were excluded from the study, and generalisations to these patient groups cannot be made. Not all targeted individuals were included in the intervention group, either because they could not be reached or they opted out, and their respective comparators were then not included.^9^ The participants were enrolled during regular visits, which are scheduled at least yearly, so the 18 months before intervention should be enough for most eligible participants. One could argue that patients who agreed to participate and had a least one visit in connection with the intervention represent a group with more commitment to their treatment and less prone to relapse at baseline than patients in general, creating selection bias. However, the number of admissions was comparable between the intervention group and the comparison group in the 18 months before intervention, suggesting similar illness characteristics. We lacked information on confounders such as socioeconomic status, illness duration and substance misuse. However, their potential impact on the results was limited by the randomisation process. All intervention visits were conducted by the same person, assuring consistency across visits. We used hospital admissions and switch to an LAI as proxies for relapse and for patient involvement respectively, which may be questioned. However, these outcomes were readily available hard outcomes in the medical charts,

### Clinical and research implications

A combination of psychoeducation and motivational interviewing may be a valuable tool in the care of people with schizophrenia and schizoaffective disorder, stimulating their involvement in treatment and reducing the frequency of readmissions, but further study is needed to corroborate the findings of this study.

## Data Availability

Data associated with the manuscript is available with the corresponding author.

## About the authors

**Gabriella Bröms**, MD, PhD, is a post-doc in the Department of Medicine, Solna, Karolinska Institutet, Stockholm, Sweden. **Lindah Cahling**, is a specialist nurse with PRIMA Child and Adult Psychiatry, Stockholm, Sweden. **Anders Berntsson**, is a psychiatrist and head of clinic at PRIMA Child and Adult Psychiatry, Stockholm, Sweden. **Lars Öhrmalm**, PhD, is a psychiatrist with PRIMA Child and Adult Psychiatry, and a researcher in the Department of Medicine, Solna, Karolinska Institutet, Stockholm, Sweden.

## References

[1] Weiden PJ, Kozma C, Grogg A, Locklear J. Partial compliance and risk of rehospitalization among California Medicaid patients with schizophrenia. Psychiatr Serv 2004; 55: 886–91. 15292538 10.1176/appi.ps.55.8.886

[2] Weiden PJ. Understanding and addressing adherence issues in schizophrenia: from theory to practice. J Clin Psychiatry 2007; 68(suppl 14): 14–9. 18284273

[3] Kirson NY, Weiden PJ, Yermakov S, Huang W, Samuelson T, Offord SJ, Efficacy and effectiveness of depot versus oral antipsychotics in schizophrenia: synthesizing results across different research designs. J Clin Psychiatry 2013; 74(6): 568–75. 23842008 10.4088/JCP.12r08167

[4] Potkin S, Bera R, Zubek D, Lau G. Patient and prescriber perspectives on long-acting injectable (LAI) antipsychotics and analysis of in-office discussion regarding LAI treatment for schizophrenia. BMC Psychiatry 2013; 13: 261. 24131801 10.1186/1471-244X-13-261 PMC3819472

[5] Waddell L, Taylor M. Attitudes of patients and mental health staff to antipsychotic long-acting injections: systematic review. Br J Psychiatry 2009; 195(suppl 52): S43–50. 10.1192/bjp.195.52.s43 19880916

[6] David AS. Treatment adherence in psychoses. Br J Psychiatry 2010; 197: 431–2. 21119147 10.1192/bjp.bp.110.083022

[7] Zygmunt A, Olfson M, Boyer CA, Mechanic D. Interventions to improve medication adherence in schizophrenia. Am J Psychiatry 2002; 159: 1653–64. 12359668 10.1176/appi.ajp.159.10.1653

[8] Staring AB, Van der Gaag M, Koopmans GT, Selten JP, Van Beveren JM, Hengeveld MW, Treatment adherence therapy in people with psychotic disorders: randomised controlled trial. Br J Psychiatry 2010; 197: 448–55. 21119150 10.1192/bjp.bp.110.077289

[9] Cahling L, Berntsson A, Bröms G, Öhrmalm L. Perceptions and knowledge of antipsychotics among mental health professionals and patients. BJPsych Bull 2017; 41: 254–9. 29018549 10.1192/pb.bp.116.055483 PMC5623883

[10] Miller WR, Rollnick S. Motivational Interviewing: Preparing People for Change (2nd edn). Guilford Press, 2002.

[11] Barkhof E, Meijer CJ, de Sonneville LM, Linszen DH, de Haan L. The effect of motivational interviewing on medication adherence and hospitalization rates in nonadherent patients with multi-episode schizophrenia. Schizophr Bull 2013; 39: 1242–51. 24072808 10.1093/schbul/sbt138 PMC3796095

[12] Heres S, Hamann J, Kissling W, Leucht S. Attitudes of psychiatrists toward antipsychotic depot medication. J Clin Psychiatry 2006; 67: 1948–53. 17194274 10.4088/jcp.v67n1216

